# Patient Payment and Unhealthy Behavior: A Comparison across European Countries

**DOI:** 10.1155/2017/2615105

**Published:** 2017-02-05

**Authors:** Reza Rezayatmand, Milena Pavlova, Wim Groot

**Affiliations:** ^1^Health Management and Economics Research Center, Isfahan University of Medical Sciences, Isfahan, Iran; ^2^Department of Health Services Research, CAPHRI, Maastricht University Medical Centre, Faculty of Health, Medicine and Life Science, Maastricht University, Maastricht, Netherlands; ^3^Top Institute for Evidence-Based Education Research (TIER), Maastricht University, Maastricht, Netherlands

## Abstract

*Introduction*. Prior research has documented that unhealthy behaviors result in greater health care use and greater health care costs. However, there are few studies on out-of-pocket expenditure paid by those engaging in unhealthy behaviors. We provide cross-country evidence on the association of smoking, alcohol consumption, and obesity with health care use and health care cost as well as out-of-pocket payments among the elderly in Europe.* Method*. Using SHARE dataset for 13 European countries, the study uses a sequential logit model to analyze use and payments for outpatient and inpatient health care service in addition to a two-part model for the analysis of use and payments for prescribed drugs.* Results*. Former smoking is associated with a higher rate of health care use. However, current smoking is associated with lower health care use. Former smoking is also associated with paying higher amount of out-of-pocket payments. Alcohol consumption is associated with lower health care use.* Conclusion*. We do not find systematic evidence that unhealthy behaviors among elderly (50+) are associated with more utilization of health care and more out-of-pocket payments. The results can be of interest for policies that aim to make people more responsible toward their health behaviors.

## 1. Introduction

Prior research has documented that unhealthy behaviors result in greater health care use and consequently greater health care costs [1–4]. In a solidarity-based health care system, like in most European countries, the burden of the costs is incurred by all individuals (citizens), who contribute to the system funding and not only by those who cause the costs. This issue has been criticized, as it seems unfair that the costs of a choice for an unhealthy behavior are paid for by those who have chosen a healthy lifestyle [5]. Thus, there is an ongoing debate about incorporating some elements of individual responsibility in the health care system and providing incentives for a better health-related behavior [6, 7]. For instance, some studies suggest a carrot and stick approach for this purpose: carrot like the discount in premium for those who have a healthier lifestyle and a stick like a copayment for those who have a medical problem due to their unhealthy lifestyle. There are also some other suggestions like waiting list for those who have an unhealthy lifestyle [8].

This debate is based on the expectation that unhealthy behaviors increase use and costs of health care. Many studies have investigated the correctness of this assumption [9–17]. Regarding smoking, previous studies [9–11] have shown that current smokers use outpatient care less often than never smokers, but, if anything, the number of physician visits is higher for current smokers than for never smokers. Regarding inpatient care, previous studies report more hospitalization either by current smokers [9] or by former smokers [11] or only among female former smokers [10] compared to never smokers. Some studies have looked at other health-related behaviors too, though there are fewer studies on this than on the effect of smoking. For instance, one study [10] has shown that obesity and overweight are strongly related to a higher probability of outpatient visits in both genders and with the probability of hospitalization in women. Another study [17] has found that, among 50- to 84-year-old women in England, around one in eight hospital admissions are likely to be attributable to overweight or obesity, which means 420,000 extra hospital admissions and two million extra days spent in hospital annually.

Many studies have shown that smoking, obesity, overweight, and alcohol consumption lead to more medical expenditure at least in the short run [9, 12, 13, 18]. However, there is no such consensus about the long-run effects of unhealthy behaviors on health care costs, particularly in case of smoking. Some studies indicate that the extra costs caused by smokers are compensated for by their higher mortality rate at earlier stages of life than nonsmokers, concluding that smoking cessation would lead to increased health care costs due to nonsmoker longevity [12, 19]. In contrast, other studies show that in spite of smokers having a shorter lifespan than nonsmoker, the total lifetime medical expenditure is still higher among smokers, and it increases with the amount of smoking [20].

Most previous studies have commonly used a top-down approach using data at the population level. At the same time, these studies have mostly looked at the cost of unhealthy behaviors from a societal perspective. There are few studies investigating out-of-pocket expenditure paid by those who engaged in unhealthy behaviors. If it can be shown that those who engage in unhealthy behavior and use the health care system more often also pay more out of their pocket, it means that they have already paid some of their way.

The aim of our study is to address the following two questions: first, whether or not an unhealthy lifestyle is associated with more utilization of health care and, second, whether the extra cost, if any, is paid out-of-pocket by those who engage in an unhealthy behavior or is paid out of a collective pocket. In this study, we look at daily smoking, body mass index (BMI), and heavy drinking to proxy health-related behaviors. We use individual-level data on the utilization of outpatient and inpatient care as well as data on the utilization of medication from 13 European countries. Specifically, we use data from the second wave of the Survey of Health, Aging and Retirement in Europe (SHARE), which targets the elderly population (aged 50+). A sequential logit model is used for the analysis of use and payments for outpatient and inpatient health care service in addition to a two-part model for the analysis of use and payments for prescribed drugs. We contribute to previous research by providing evidence on the internal medical costs of unhealthy behavior among elderly in a broad range of European countries. The results can be of interest for developing policies that aim to make people more responsible toward their health behavior.

## 2. Methods

### 2.1. Study Population

To study the association of health-related behavior with health care use and payments among the elderly in European countries, we use data from the second wave of the Survey on Health, Aging and Retirement in Europe (SHARE, release 2.5.0). SHARE is a multidisciplinary and cross-national panel dataset with microlevel data on health, socioeconomic status, and social and family networks. The survey is conducted every two years, starting from 2004, using nationally representative samples of individuals aged 50 or over in Europe. The most recent wave for our analysis was the second wave of SHARE which included our questions of interest about out-of-pocket expenditure while including more countries than the first wave. The second wave was conducted in 2006-2007 in 14 European countries, namely, Austria, Germany, Sweden, Netherlands, Spain, Italy, France, Denmark, Greece, Switzerland, Belgium, Czech Republic, Poland, and Ireland [21]. We exclude Ireland because the imputed variables generated by SHARE were not available for that country. Thus, overall, our sample size consists of 33281 respondents from 13 countries.

### 2.2. Out-of-Pocket Expenditures

In the second wave of SHARE, one part of the questionnaire was devoted to out-of-pocket expenses where the following question was asked: “not counting your health insurance premium or reimbursements from employers, about how much did you pay out-of-pocket for your outpatient care in last twelve months?” The same question was asked for inpatient care and prescribed drugs. The respondents were instructed to consider out-of-pocket payments as every expense that is not covered by their health insurance:

- For outpatient care: all expenses for consultations for all health professionals including dentists, for all labs, exam, or therapies prescribed by doctor, and for outpatient surgery
- For inpatient care: all expenses for staying in medical, surgical, psychiatric or in any other specialized wards
- For their prescribed drugs not including self-medication or drugs not prescribed by a doctor. For these variables, we use imputed values generated by SHARE. For each variable, five imputed values were provided. The median of the imputed values was used. All amounts in local currencies were converted to Euro using nominal exchange rate corresponding to the year of interview.

### 2.3. Analytical Model

**Figure 1:**
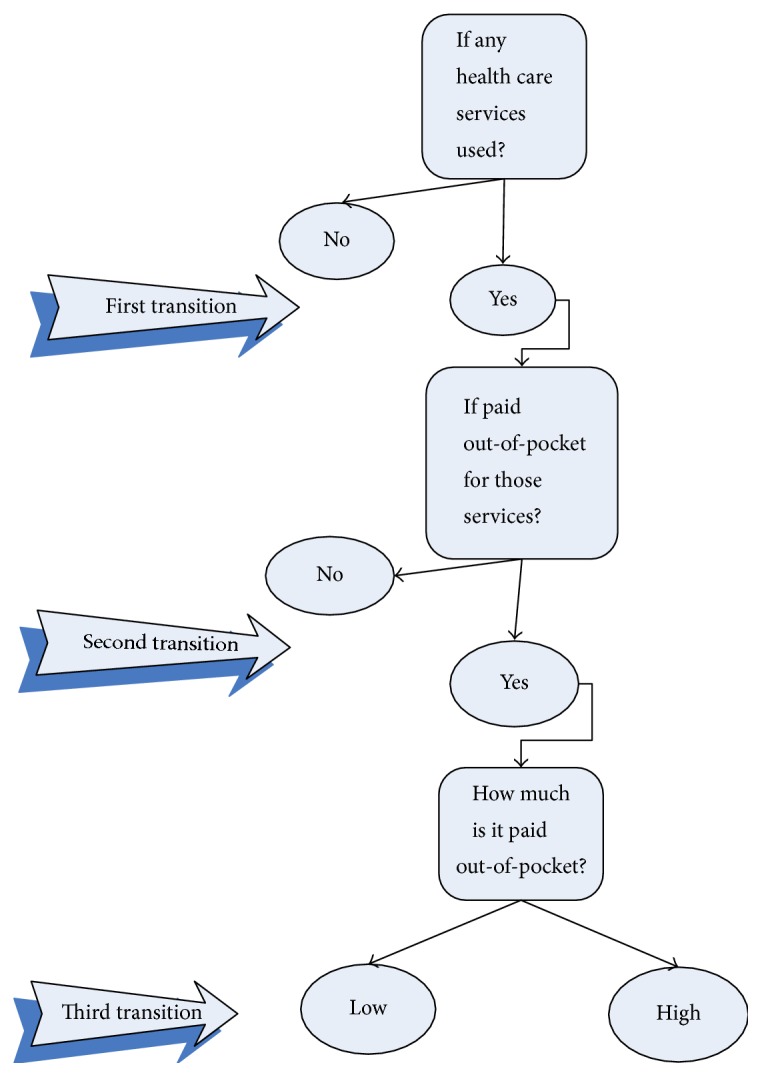
Sequential logit for outpatient and inpatient out-of-pocket payment. Low: lower than the median for out-of-pocket payments for those services in the country; high: higher than the median for out-of-pocket payments for those services in the country.

**Figure 2:**
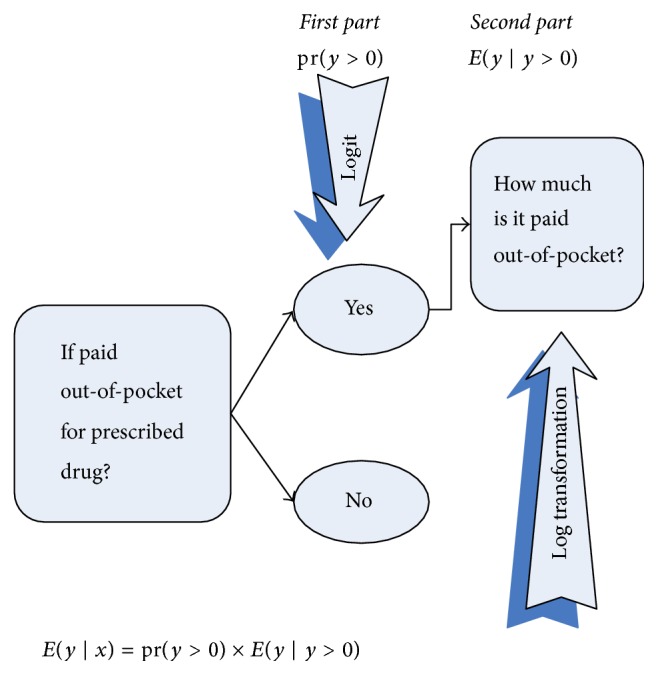
Two-part model for prescribed drug.

Out-of-pocket payments are an outcome of a consecutive process. At the first sequence, the service should be used, then the payment should be made, and subsequently the amount of payment should be determined. Therefore, a sequential logit model was used to study the association between health behaviors (i.e., smoking, alcohol consumption, and obesity) and the odds ratios of passing through each transition from utilization of outpatient and inpatient care to the size of out-of-pocket payments for these services (Figure 1). For prescribed drugs, we were not able to distinguish between the service use and the payment for that service as we did for outpatient and inpatient care. Thus, a two-part model was used to model the probability of payment at first and then the amount of payment for those who paid (Figure 2). Three models were estimated: for outpatient care, for inpatient care, and for prescribed drugs. The analyses were done jointly for all countries as well as separately for each country.

### 2.4. Health Behaviors

Indicators of smoking, alcohol consumption, and preobesity (overweight) or obesity were included in the models as independent variables. For smoking, current and former daily smokers were compared with those who had never smoked daily for a period of at least one year (reference group). Alcohol consumption was assessed using the question about the frequency of excessive alcohol use in the last three months. Excessive alcohol use was defined as drinking more than two glasses of any alcoholic beverage (e.g., beer, cider, wine, spirits, or cocktails) almost every day or five or six times a week. Respondents were categorized into three groups: excessive alcohol users, not excessive alcohol users, and those who had never drank in the past 3 months (reference group). Weight problems were measured on the basis of body mass index (BMI) calculated from self-reported height and weight. Respondents were classified into three categories: obese (BMI ≥ 30), preobese (25 ≤ BMI < 30), and those with BMI < 25 (reference group). It should be noted that obesity and overweight are not health behaviors but the results of health-related behaviors such as nutrition and physical activity. In this study, we use them to proxy those health-related behaviors.

### 2.5. Other Explanatory Variables

All models controlled for age, gender, living with spouse or partner, years of education, household size, annual household net income quartile, the number of chronic diseases, the number of symptoms, and a dummy variable indicating the self-perceived health status (very good or excellent and less than very good or excellent). Previous studies have shown that risk tolerance is a significant predictor of unhealthy behavior such as smoking and drinking [22]. At the same time, risk aversion can affect health care utilization of those who engaged in unhealthy behaviors. So the model should include a measure of risk aversion. SHARE asks the individual when deciding about making an investment if they are willing to take a substantial, above average, or average financial risk to earn substantial, above average, or average return, or they are not willing to take any financial risk at all. We use the answer to this question as a proxy for risk aversion. However, the reference group is those who said that this question is not applicable to them to represent the most risk adverse individuals.

## 3. Results

### 3.1. Descriptive Statistics

The sample characteristics are presented in Table 1. It should be noted that all results presented below refer to the elderly population (aged 50+). Of all participants, 20% are current daily smokers, 28% former daily smokers, and 52% never smokers. Greece and France have the highest and the lowest prevalence of current smokers (29% and 14%, resp.). Netherlands and Greece have the highest and the lowest proportion of former smokers (40% and 17%, resp.). Austria and Netherlands have the highest and the lowest proportion of never smokers (65% and 37%, resp.). Of all participants, nearly 7% are heavy drinkers, 62% occasional drinkers, and 31% never drinkers. The highest prevalence of heavy drinking is reported in Netherlands and Belgium (11%), and the lowest prevalence is in Sweden (1.5%). Denmark and Sweden have the highest proportion of occasional drinkers (about 81%), and Spain has the lowest proportion (37%). Spain has the highest proportion of never drinkers (57%), and Denmark has the lowest one (8%). Weight problems appear the most prevalent unhealthy behavior as only 37% of all respondents report a normal weight. About 19% of all respondents are obese and 42% are preobese (overweight). Obesity is most prevalent in Poland and Spain (nearly 25% of the participants in these countries). Preobesity is most prevalent in Greece (47.5%). Switzerland has the highest number of individuals whose weight is in the normal range while Spain has the lowest number (47.5% versus 27%).

The annual out-of-pocket payments, if any, for outpatient care, inpatient care, and prescribed drugs are, on average, €320, €509, and €200, respectively. The highest amount of out-of-pocket payments for outpatient care are reported in Switzerland (€700), for inpatient care in Greece (€1878), and for prescribed drugs in Poland (€333). The lowest amounts of out-of-pocket payments for these three services are reported in the Czech Republic. The sample characteristics regarding sociodemographic and health status are presented in Appendix A1 in Supplementary Material available online at https://doi.org/10.1155/2017/2615105.

### 3.2. Outpatient Out-of-Pocket Payments

Table 2 presents the results of a sequential logit for out-of-pocket payments for outpatient care. As mentioned in Methods, the model estimates the odds ratio of passing three transitions: whether or not the service is used, whether or not an out-of-pocket payment is made, and whether a high or a low amount is paid.

As depicted in Table 2, current smokers are less likely (OR = 0.72) than never smokers to use outpatient care. However, there is no statistically significant difference between current smokers and never smokers in terms of out-of-pocket payments. Former smokers are more likely (OR = 1.15) than never smokers to use outpatient care. They are also more likely (OR = 1.10) to pay higher amounts than never smokers if any out-of-pocket payment is made, although the odds ratio of out-of-pocket payments is not statistically significant. Heavy drinkers are less likely (OR = 0.73) than never drinkers to use outpatient care. For out-of-pocket payments, the results do not show statistically significant differences between heavy drinkers and never drinkers. However, light drinkers are more likely (OR = 1.17) to pay out of pocket in case of using outpatient care, although they are as likely as never drinkers to use outpatient care. The findings related to obesity and preobesity do not appear statistically significant.

### 3.3. Inpatient Out-of-Pocket Payments

Table 3 presents, in turn, the odds ratios for whether or not an inpatient care service is used, for whether or not an out-of-pocket payment is made, and for whether a high or a low amount is paid.

Former smokers are more likely (OR = 1.37) to use inpatient care than never smokers but when they use the service, there is no statistically significant difference in terms of out-of-pocket payments. In contrast, both heavy and light drinkers are less likely (OR = 0.53 and OR = 0.70, resp.) than never drinker to be hospitalized. Obese and preobese individuals are also less likely (OR = 0.88 and OR = 0.90, resp.) to use inpatient care than those with normal weight.

### 3.4. Prescribed Drug Out-of-Pocket Payment

Table 4 presents the results of the two-part model for the out-of-pocket payments for prescribed drugs. As previously noted, the first part is a logit model estimating the odds of out-of-pocket payment. The second part is a log-linear model estimating the amount of out-of-pocket payments.

Current smokers are less likely than never smokers (OR = 0.81) to make out-of-pocket payments for prescribed drugs. The odds of an out-of-pocket payment are not statistically different for former and never smokers. However, former smokers, on average, incur 6% more out-of-pocket expenditure than never smokers. The odds of out-of-pocket payments are not statistically significant for heavy and light drinkers; but if any payment has been made, light and heavy drinking are associated with 21% and 26% decrease in the amount of out-of-pocket payments compared with never drinkers. Those who are preobese are more likely to pay out of pocket for prescribed drugs. However, it has no effect on the amount of payment. In contrast, being obese is not related to the odds of an out-of-pocket payment, while, in case of payment size, it increases the amount of out-of-pocket payments by 7%.

In order to show the bivariate associations of smoking, alcohol consumption, and obesity with our dependent variables of interest, we have also reported the results of uncontrolled models as Appendixes A2–A4.

### 3.5. Per-Country Analysis

The stratified regression analysis by country shows some differences compared with the aggregate results presented above. Specifically, as depicted in Table 5, light drinkers in Germany (OR = 0.61), Spain (OR = 0.71), and Poland (OR = 0.68) are less likely, but those who in Greece (OR = 1.39) are more likely to use outpatient care than never drinkers. In France and Poland, overweight appears to be associated with higher odds (OR = 1.83 and OR = 1.47, resp.) of outpatient care use. However, in Sweden, obesity is associated with lower odds of outpatient care use (OR = 0.62). In contrast, in Poland, it is associated with higher odds of outpatient care use (OR = 0.62). The results of per-country analysis also show that current smokers in Austria, Netherlands, and Poland are less likely (OR = 0.37, OR = 0.71, and OR = 0.58, resp.) and former smokers in Italy and Belgium are more likely (OR = 1.36 and OR = 1.24, resp.) to pay out-of-pocket for outpatient care. In Netherlands and Switzerland, heavy drinkers are more likely (OR = 1.73 and OR =2.62, resp.) to pay out-of-pocket, while, in Greece and Belgium, they are less likely (OR = 0.58 and OR = 0.73, resp.) to do so. In Sweden and Belgium, preobesity is associated with higher odds of out-of-pocket payment for outpatient care (OR = 1.62 and OR = 1.37, resp.). However, in Italy, Denmark, and Czech Republic, obesity is associated with lower odds of out-of-pocket payment (OR = 0.77, OR = 0.75, and OR = 0.66, resp.). In contrast, in Belgium, it is associated with higher odds of out-of-pocket payments (OR = 1.37). Regarding the amount of out-of-pocket payments, the only statistically significant results are observed in Denmark for former smokers (OR = 1.53) and in Switzerland for light drinkers (OR = 0.59).

We also observe some country-specific results regarding prescribed drugs. As depicted in Table 6, the odds of out-of-pocket payments for prescribed drugs appear to be statistically significant not only for smoking and being preobese but also for other behaviors in some countries. Specifically, in Greece and Switzerland, light drinking is associated with higher odds (OR = 1.22 and OR=1.61, resp.) but in Czech Republic and Poland with lower odds (OR = 0.70) of out-of-pocket payments for prescribed drugs. In Greece, Belgium, and Poland, obesity is associated with higher odds of out-of-pocket payments for prescribed drugs (OR = 1.31, OR = 1.54, and OR = 1.72, resp.). Regarding the amounts of out-of-pocket payments for prescribed drugs, the results are not statistically significant for current smoking and obesity in aggregate models. However, as depicted in the second part of Table 6, in Germany, current smoking is associated with a higher amount of out-of-pocket payments. In Germany and Poland, preobesity is associated with a higher amount of out-of-pocket payments, while, in Spain, it is associated with lower amount of out-of-pocket payments.

## 4. Discussion

Using individual-level data from 13 European countries, we employ a sequential logit model to investigate how the utilization of outpatient and inpatient care and out-of-pocket payments for them is associated with health-related behavior. We also use a two-part model to study the association between out-of-pocket payments for prescribed drugs and health-related behavior. As our sample only includes individuals aged 50 and over, our results cannot be generalized to other age groups. Our study is also not able to distinguish between those services that require out-of-pocket payments and those that do not. Instead, we use a retrospective approach among those who used a service to investigate to what extent out-of-pocket payment for those services can be associated with individuals' unhealthy behavior. In this study, we use self-reported BMI to proxy corresponding health-related behaviors such as nutrition and physical activity. It should be also mentioned that, due to the low number of observations in the last transition, per-country analysis was not possible for inpatient care. Despite these limitations, we contribute to current research by adding new findings to the sparse evidence on the internal costs of an unhealthy behavior, that is, the cost which is borne by the individuals themselves. These costs can be of more importance for policies about holding people more responsible toward their health-related behavior, as they are those costs that are internally perceived.

### 4.1. Health-Related Behavior and Health Care Utilization

Our results cannot confirm that unhealthy behavior is systematically associated with more health care utilization. However, the common pattern in most countries included in our study is a higher rate of inpatient use by former smokers. Previous studies have also shown a higher rate of hospitalization for former smokers but, in contrast to our study, also for current smokers [9, 18]. Regarding outpatient care, our findings comply with previous findings. Sloan et al. [18] also show a lower physician visits rate for current smokers at age 50 plus than never smokers in the United States. Izumi et al. [9], having considered current and former smokers together as one group of smokers, find that Japanese smokers use outpatient care less often than never smokers. Their study was not limited to the elderly. Wacker et al. [11], using data from Germany, find that current smokers are less likely to use outpatient care but if anything the rate of physician visits is higher among them compared to never smokers.

Lower utilization by current smokers has been attributed to their lower concerns about their health status or the fact that smokers might be less risk adverse individual [9]. It means that smokers usually seek health services at a later stage of their diseases or even after the time that a disease had forced them to quit smoking, which can somehow explain the higher rate of health care utilization among former smokers. In this study, we have controlled for risk aversion. The proxy of risk aversion, which we use, indicates individual's willingness to take financial risk. We are aware of the fact that a health risk might be perceived completely differently from a financial risk. Thus, this might not be the best variable controlling the risk preference, but this was the only available option given in the dataset. Lower health care utilization by current smoker can be also explained by the fact that the health consequence of smoking appears some years later. The same argument will be valid for other unhealthy behaviors too.

Regarding other health behaviors, the results were either not statistically significant or showing a negative association between outpatient and inpatient care. Few countries are exceptional cases, for instance, in Greece for light drinkers or in France and Poland for those who are preobese. Vals et al. [10], using data from Estonia, have found that light drinking, obesity, and preobesity are associated with more use of health care services.

The most common pattern among the elderly in the included countries is that alcohol consumption is associated with lower use of health care services. One explanation for excessive alcohol use is that not heavy drinking might have some health reason, meaning that those who drink heavily might be either less concerned about their health or healthier than those who do not drink [10]. However, we control for three indicators of health status, namely the number of chronic diseases, the number of symptoms and self-perceived health status. It means that for two individuals at the same health status (regarding these indicators), the heavy drinker is less likely to use outpatient and inpatient care.

### 4.2. Health-Related Behavior and Out-of-Pocket Payments

The results do not show a systematic association between health behaviors and out-of-pocket payments. The most repeating pattern among countries is a positive association between light drinking and out-of-pocket payments for outpatient care, as well as a negative association between current smoking and out-of-pocket payment for prescribed drugs. With regard to the size of out-of-pocket payments, the common pattern which is repeated in most countries (8 of 13 countries included in this study) is that alcohol consumption is associated with lower out-of-pocket payments for prescribed drugs. In contrast, whenever statistically significant (in Spain, Greece, and Belgium), the results show that ex-smoking is associated with a higher amount of out-of-pocket payment for prescribed drugs.

Less often out-of-pocket payments for prescribed drugs are explained by less use of outpatient care among current smokers in the respective country. However, it can also be observed in countries where the lower odds of outpatient care use for current smokers are not statically significant (i.e., Netherlands and France). At the same time, although, in Belgium, current smokers are less likely to use outpatient care, the lower odds of out-of-pocket payments for prescribed drugs are not statistically significant. The higher odds of out-of-pocket payments for outpatient care in case of light dinking are quite challenging to be explained. They are observed in countries (Germany, Netherlands, Italy, Switzerland, and Czech Republic) which are very different in terms of both health care financing and the prevalence of light drinking. In addition, these results are not due to more utilization of outpatient care among light drinkers, as we see less use of outpatient care by light drinkers. One explanation might be that although light drinkers generally use less outpatient care, while using, they will use kinds of outpatient services which usually require higher out-of-pocket payment. In addition, our data looked backward only three months for alcohol consumption. Thus, the reason of abstaining from alcohol might be the health problem. It means that abstainers in the last three months might have more sever health problems which lead them to quit drinking and can explain more out-of-pocket payment among them.

### 4.3. Relevance of the Country Context

For some health behaviors, the association between health behavior and out-of-pocket payments shown in the aggregated models is driven by a single country. For instance, the association between preobesity and out-of-pocket payments in aggregate model is only driven by Poland or the association between ex-smoking and the amount of out-of-pocket payment is only driven by Denmark. This suggests that the interpretation of our results on out-of-pocket payments should be based on the countries' contextual factors. For example, in Poland, the share of out-of-pocket expenditure on total pharmaceutical expenditure is the highest among OECD countries [23]. Thus, the basic pharmaceutical package is very limited. In addition, 41% of the Polish individuals in our sample are preobese. As a result, the observed association between preobesity and out-of-pocket payments for prescribed drugs may just reflect higher out-of-pocket expenditures for pharmaceutical by the elderly in general. In Denmark, there is no official charge for outpatient visits provided that a predetermined pathway is followed by patients. However, if individuals prefer more choice of health care providers, they have to pay out-of-pocket. Thus, the higher out-of-pocket payments for outpatient services by ex-smokers that we find for Denmark might be due to preferences of elderly ex-smokers to choose their doctor on their own. We recognize, however, that this is only a hypothetical explanation, which needs to be tested.

## 5. Conclusion

Having compared 13 European countries, we do not find systematic evidence that unhealthy behavior among elderly is associated with more utilization of health care and more out-of-pocket payments. The most consistent pattern among the elderly in the included countries appears in case of smoking. It shows that former smoking is associated with higher rate of health care use. In contrast, current smoking is associated with lower rate of health care use. These findings can have important policy implication regarding stick and carrot approach for individual responsibility for health. It shows that people at the time of engaging in an unhealthy behavior (i.e., current smoking and heavy drinking) are not using more health care than those who do not engage in respective behavior. Thus, using a stick like copayment might not encourage them to quit. However, when people are in need of health care, resulting in more use of health care, they are most probably not engaging in unhealthy behavior (i.e., ex-smoking). Thus, using a stick like copayment would limit access to health care for those who need it which contradicts the principles of equity and solidarity.

Looking at the contextual factors in each country is crucial. Our findings per country can be of use for further studies on the causes of cross-country differences in out-of-pocket payments.

## Supplementary Material

Supplementary Material provides more detailed information about the results. Appendix A1 shows detailed information on the sample characteristics regarding sociodemographic and health status. Appendix A2-4 report the results of uncontrolled models for analysis done in the paper for outpatient and inpatient out-of-pocket payment, as well as for prescirbed drug out-of-pocket payment.

## Figures and Tables

**Table 1:** *Sample Characteristics*. Health-related behavior and out-of-pocket (OOP) payments.

	Austria	Belgium	Czech Republic	Denmark	France	Germany	Greece	Italy	Netherland	Poland	Spain	Sweden	Switzerland	Total
*Daily smoking *														
Current (%)	15.4	17.5	21.2	26.4	13.8	16.8	28.9	17.0	22.3	25.7	15.4	14.5	18.8	19.8
Former (%)	20.2	31.3	18.8	35.2	28.8	27.0	17.1	26.0	40.4	28.4	21.2	36.1	25.2	27.9
Never (%)	64.4	51.3	60.0	38.4	57.4	56.2	54.1	57.0	37.3	45.9	63.4	46.4	56.0	52.3
*n*	1199	3139	2801	2570	2922	2529	3231	2969	2605	2448	2200	2713	1450	32776

*Alcohol consumption*														
Heavy (%)	3.5	10.8	4.0	10.2	10.2	4.7	5.1	8.6	10.8	1.7	5.6	1.4	6.9	6.6
Light (%)	65.1	68.1	55.7	81.6	63.6	72.9	52.7	45.7	65.5	50.5	37.1	81.0	78.9	62.3
Not at all (%)	31.3	21.2	40.4	8.2	26.3	22.4	42.2	45.7	23.7	47.9	57.3	17.6	14.3	31.1
*n*	1331	3138	2797	2558	2905	2539	3236	2975	2619	2448	2217	2705	1445	32913

*Body Mass Index*														
BMI ≥ 30	24.5	18.9	24.2	14.6	16.0	17.7	20.2	18.5	14.9	25.7	25.0	16.0	12.8	19.0
25 ≤ BMI < 30	38.4	40.5	46.8	38.3	38.1	44.8	47.5	44.5	41.4	41.0	47.4	39.4	37.1	42.3
18.5 ≤ BMI < 25	35.0	38.3	28.2	44.8	43.8	36.7	31.7	36.1	42.5	31.9	26.6	42.1	47.4	37.2
BMI < 18.5	2.2	2.2	0.7	2.4	2.2	0.8	0.6	0.9	1.3	1.4	1.1	2.5	2.7	1.5
*n*	1160	3070	2769	2549	2840	2491	3215	2912	2526	2425	2016	2682	1433	32088

*Outpatient OOP *(*€*)^1^														
Mean	330	176	84	392	247	137	322	605	378	123	489	273	698	320
SD	546	601	155	950	542	479	619	1432	788	248	974	2631	2056	1477
*n*	178	1866	246	923	391	1477	1450	1010	446	158	207	2306	900	11558

*Inpatient OOP *(*€*)^1^														
Mean	229	623	75	918	476	159	1878	1639	233	127	934	76	1128	509
SD	485	2227	94	780	1279	262	3252	3125	290	186	1438	120	3027	1763
*n*	235	407	34	5	125	330	134	46	12	40	24	331	110	1833

*Prescribed drug OOP *(*€*)^1^														
Mean	199	282	61	242	116	115	185	260	135	333	166	179	204	200
SD	291	395	105	342	201	216	303	1215	213	2914	414	2154	455	1226
*n*	1070	2590	2184	1709	912	1825	2225	1973	376	1958	608	2030	756	20216

^1^Out-of-pocket payment if made.

**Table 2:** Results of sequential logit for outpatient out-of-pocket payment.

	Either used or not			Either paid or not			Either paid high or low amount		
	OR	95% CI		OR	95% CI		OR	95% CI	
		L	U		L	U		L	U
*Daily smoking*									
Current smoker	0.72^*∗∗*^	0.66	0.79	0.93^+^	0.86	1.01	1.01	0.90	1.14
Former smoker	1.15^*∗∗*^	1.05	1.26	1.07^+^	0.99	1.15	1.10^*∗*^	1.002	1.21
*Alcohol consumption*									
Not excessive alcohol use	0.97	0.89	1.06	1.17^*∗∗*^	1.09	1.26	0.94	0.84	1.04
Excessive alcohol use	0.73^*∗∗*^	0.63	0.85	1.02	0.90	1.17	1.02	0.84	1.24
*Body mass index*									
Preobese	1.06	0.98	1.15	1.02	0.95	1.09	0.98	0.89	1.07
Obese	0.98	0.88	1.09	0.93^+^	0.85	1.01	1.03	0.91	1.15
Age	1.01^*∗∗*^	1.01	1.01	0.99^*∗∗*^	0.99	1.00	1.00	1.00	1.01
Female	1.36^*∗∗*^	1.26	1.47	1.17^*∗∗*^	1.10	1.26	1.11^*∗*^	1.02	1.22
Living with partner	0.94^*∗*^	0.89	1.00	0.95^*∗*^	0.91	1.00	1.03	0.96	1.10
Years of education	1.02^*∗∗*^	1.01	1.03	1.03^*∗∗*^	1.02	1.04	1.03^*∗∗*^	1.02	1.04
*Willingness to take financial risk*									
Substantial financial risk	1.17	0.81	1.71	0.86	0.60	1.22	1.22	0.77	1.93
Above average financial risk	1.25^*∗*^	1.03	1.50	1.38^*∗∗*^	1.13	1.68	1.16	0.96	1.40
The average financial risk	1.12^+^	0.99	1.26	1.33^*∗∗*^	1.20	1.47	1.21^*∗∗*^	1.06	1.38
Not willing to take any risk	1.04	0.95	1.13	1.05	0.97	1.13	1.00	0.90	1.10
Household size	0.91^*∗∗*^	0.88	0.95	0.95^*∗*^	0.92	0.99	0.98	0.93	1.04
*Annual household net income *									
1th quartile	0.80^*∗∗*^	0.71	0.89	0.96	0.87	1.06	0.80^*∗∗*^	0.69	0.91
2th quartile	0.99	0.89	1.10	1.13^*∗∗*^	1.03	1.24	0.85^*∗∗*^	0.76	0.96
3th quartile	1.08	0.98	1.19	1.06	0.97	1.15	0.91^+^	0.81	1.01
*Household net asset*									
1th quartile	0.88^*∗*^	0.79	0.98	0.84^*∗∗*^	0.76	0.92	0.87^*∗*^	0.76	0.98
2th quartile	0.97	0.88	1.07	0.93^*∗*^	0.85	1.01	0.91	0.81	1.02
3th quartile	0.97	0.88	1.07	1.02	0.94	1.11	0.99	0.88	1.10
Self-perceived health	1.60^*∗∗*^	1.48	1.73	1.07^+^	0.99	1.16	1.35^*∗∗*^	1.23	1.49
Number of chronic diseases	1.97^*∗∗*^	1.87	2.07	1.00	0.97	1.03	1.14^*∗∗*^	1.09	1.18
Number of symptoms	1.27^*∗∗*^	1.23	1.32	1.02^*∗*^	1.00	1.04	1.10^*∗∗*^	1.07	1.13
*Country dummies* ^1^									
Austria	1.88^*∗∗*^	1.48	2.38	0.81^+^	0.65	1.01	1.38	0.91	2.08
Belgium	2.87^*∗∗*^	2.40	3.44	7.18^*∗∗*^	6.26	8.24	1.01	0.80	1.26
Czech republic	2.29^*∗∗*^	1.92	2.74	0.44^*∗∗*^	0.37	0.52	0.88	0.63	1.23
Denmark	1.11	0.95	1.30	2.27^*∗∗*^	1.96	2.63	1.10	0.85	1.42
France	3.98^*∗∗*^	3.25	4.87	0.69^*∗∗*^	0.59	0.81	0.94	0.70	1.26
Germany	2.22^*∗∗*^	1.86	2.66	6.88^*∗∗*^	5.96	7.94	2.13^*∗∗*^	1.68	2.70
Greece	1.06	0.91	1.23	5.47^*∗∗*^	4.74	6.32	1.18	0.93	1.51
Italy	1.57^*∗∗*^	1.33	1.85	3.02^*∗∗*^	2.61	3.48	1.05	0.82	1.35
Poland	0.65	0.55	0.76	0.39^*∗∗*^	0.31	0.47	1.07	0.72	1.60
Spain	1.65^*∗∗*^	1.37	1.99	0.60^*∗∗*^	0.49	0.73	1.29	0.89	1.89
Sweden	0.81^*∗∗*^	0.70	0.95	72.99^*∗∗*^	58.54	91.01	1.00	0.80	1.26
Switzerland	1.50^*∗∗*^	1.25	1.80	9.60^*∗∗*^	8.10	11.37	1.27^+^	0.99	1.64

^1^With reference to Netherlands.

^*∗∗*^Significant at 1% level, ^*∗*^significant at 5% level, and ^+^significant at 10% level.

**Table 3:** Results of sequential logit for inpatient out-of-pocket payment.

	Either used or not			Either paid or not			Either paid high or low amount		
	OR	95% CI		OR	95% CI		OR	95% CI	
		L	U		L	U		L	U
*Daily smoking*									
Current	1.01	0.91	1.12	0.85	0.65	1.11	1.06	0.77	1.44
Former	1.37^*∗∗*^	1.27	1.49	0.94	0.76	1.16	1.03	0.82	1.30
*Alcohol consumption*									
Not excessive alcohol use	0.70^*∗∗*^	0.65	0.76	1.14	0.94	1.38	0.93	0.74	1.17
Excessive alcohol use	0.53^*∗∗*^	0.45	0.62	1.38	0.90	2.13	1.04	0.63	1.72
*Body mass index*									
Preobese	0.88^*∗∗*^	0.82	0.95	0.99	0.82	1.21	0.95	0.77	1.19
Obese	0.90^*∗*^	0.82	0.99	0.86	0.68	1.09	0.93	0.71	1.23
Age	1.01^*∗∗*^	1.01	1.01	1.00	0.99	1.01	1.01^+^	1.00	1.02
Female	0.78^*∗∗*^	0.73	0.85	0.99	0.81	1.21	0.84	0.68	1.04
Living with partner	1.02	0.97	1.07	0.99	0.86	1.12	1.00	0.86	1.17
Years of education	1.01^+^	1.00	1.02	1.03^*∗*^	1.00	1.06	0.98	0.96	1.01
*Willingness to take financial risk*									
Substantial financial risk	0.77	0.49	1.21	3.58^*∗*^	1.11	11.56	0.52	0.14	1.86
Above average financial risk	1.00	0.81	1.24	1.21	0.59	2.48	1.22	0.72	2.06
The average financial risk	0.99	0.87	1.11	1.27	0.93	1.75	1.02	0.71	1.45
Not willing to take any risk	0.98	0.90	1.07	1.14	0.92	1.42	0.79^+^	0.62	1.01
Household size	0.97	0.94	1.01	1.01	0.91	1.12	1.11	0.95	1.29
*Annual household net income *									
1th quartile	0.95	0.84	1.06	1.02	0.76	1.38	0.90	0.64	1.26
2th quartile	0.99	0.90	1.10	1.16	0.88	1.52	0.73^*∗*^	0.53	0.99
3th quartile	1.05	0.95	1.16	1.29^+^	0.99	1.67	0.81	0.60	1.09
*Household net asset*									
1th quartile	0.98	0.89	1.09	0.92	0.71	1.20	0.93	0.69	1.26
2th quartile	0.92	0.83	1.02	1.07	0.82	1.39	0.83	0.62	1.11
3th quartile	0.90^*∗*^	0.82	1.00	1.00	0.77	1.30	0.82	0.61	1.11
Self-perceived health	2.09^*∗∗*^	1.88	2.32	1.01	0.76	1.35	1.10	0.82	1.49
Number of chronic diseases	1.22^*∗∗*^	1.19	1.26	1.01	0.94	1.08	1.08^+^	1.00	1.17
Number of symptoms	1.17^*∗∗*^	1.15	1.20	1.05^*∗*^	1.00	1.10	0.99	0.94	1.04
*Country dummies* ^1^									
Austria	2.45^*∗∗*^	2.01	3.00	78.60^*∗∗*^	40.63	152.05	0.84	0.25	2.77
Belgium	1.36^*∗∗*^	1.15	1.60	90.02^*∗∗*^	48.24	168.00	0.81	0.25	2.62
Czech republic	1.18^+^	0.99	1.39	1.81^+^	0.91	3.60	0.94	0.24	3.66
Denmark	1.23^*∗*^	1.03	1.48	0.25^*∗*^	0.08	0.80	2.19	0.17	28.10
France	1.14	0.96	1.35	9.15^*∗∗*^	4.92	17.04	0.97	0.29	3.27
Germany	1.41^*∗∗*^	1.19	1.68	82.51^*∗∗*^	43.81	155.41	0.92	0.28	2.98
Greece	0.57^*∗∗*^	0.47	0.69	33.01^*∗∗*^	17.26	63.14	1.01	0.30	3.42
Italy	1.02	0.86	1.21	2.93^*∗∗*^	1.50	5.74	0.69	0.19	2.55
Poland	1.01	0.85	1.21	2.46^*∗∗*^	1.25	4.87	0.67	0.18	2.55
Spain	0.93	0.77	1.13	2.19^*∗*^	1.03	4.66	0.80	0.19	3.42
Sweden	1.12	0.93	1.34	354.04^*∗∗*^	168.46	744.08	0.79	0.24	2.58
Switzerland	1.40^*∗∗*^	1.13	1.73	35.77^*∗∗*^	18.48	69.24	0.90	0.27	3.05

^1^With reference to Netherlands.

^*∗∗*^Significant at 1% level, ^*∗*^significant at 5% level, and ^+^significant at 10% level.

**Table 4:** Two part model for prescribed drug out-of-pocket payments.

	First part: logit Either paid or not			Second part: log transformation The amount of OOP (log)		
	OR	95% CI		Coeff	95% CI	
		L	U		L	U
*Daily smoking*						
Current smoker	0.81^*∗∗*^	0.75	0.87	0.00	−0.04	0.04
Former smoker	1.04	0.97	1.11	0.06^*∗∗*^	0.03	0.10
*Alcohol consumption*						
Not excessive alcohol use	1.04	0.97	1.11	−0.21^*∗∗*^	−0.25	−0.17
Excessive alcohol use	0.95	0.85	1.08	−0.26^*∗∗*^	−0.33	−0.19
*BMI*						
Preobese	1.10^*∗∗*^	1.04	1.17	0.02	−0.02	0.05
Obese	1.08^+^	1.00	1.17	0.07^*∗∗*^	0.03	0.12
Age	1.00^*∗∗*^	1.00	1.01	0.01^*∗∗*^	0.01	0.01
Female	1.33^*∗∗*^	1.25	1.41	0.03^+^	0.00	0.07
Living with partner	0.94^*∗∗*^	0.90	0.98	−0.02	−0.04	0.00
Years of education	1.03^*∗∗*^	1.03	1.04	0.01^*∗∗*^	0.01	0.02
*Willingness to take financial risk*						
Substantial financial risk	1.27	0.93	1.73	0.05	−0.13	0.22
Above average financial risk	1.18^*∗*^	1.01	1.37	−0.01	−0.10	0.07
The average financial risk	1.24^*∗∗*^	1.13	1.36	−0.04	−0.09	0.02
Not willing to take any risk	1.15^*∗∗*^	1.08	1.23	−0.02	−0.06	0.02
Household size	0.94^*∗∗*^	0.91	0.97	−0.01	−0.03	0.00
*Annual household net income *						
1th quartile	0.91^*∗*^	0.83	0.99	−0.07^*∗∗*^	−0.12	−0.02
2th quartile	1.01	0.93	1.09	−0.06^*∗∗*^	−0.11	−0.01
3th quartile	1.09^*∗*^	1.01	1.17	−0.02	−0.06	0.02
*Household net asset*						
1th quartile	1.05	0.97	1.14	−0.09^*∗∗*^	−0.13	−0.04
2th quartile	1.10^*∗*^	1.02	1.19	−0.05^*∗*^	−0.09	0.00
3th quartile	1.07^+^	0.99	1.15	−0.05^*∗*^	−0.09	−0.01
Self-perceived health	1.92^*∗∗*^	1.80	2.05	0.33^*∗∗*^	0.29	0.37
Number of chronic diseases	1.47^*∗∗*^	1.42	1.51	0.19^*∗∗*^	0.18	0.20
Number of symptoms	1.12^*∗∗*^	1.10	1.14	0.09^*∗∗*^	0.08	0.10
*Country dummies* ^1^						
Austria	31.19^*∗∗*^	25.48	38.18	0.69^*∗∗*^	0.56	0.82
Belgium	31.70^*∗∗*^	27.21	36.93	0.88^*∗∗*^	0.77	1.00
Czech republic	21.46^*∗∗*^	18.44	24.98	−0.70^*∗∗*^	−0.82	−0.58
Denmark	14.80^*∗∗*^	12.75	17.18	0.79^*∗∗*^	0.67	0.91
Germany	16.23^*∗∗*^	13.98	18.85	0.08	−0.04	0.20
Greece	18.80^*∗∗*^	16.24	21.75	0.69^*∗∗*^	0.57	0.80
France	2.30^*∗∗*^	1.99	2.66	−0.02	−0.15	0.11
Italy	12.92^*∗∗*^	11.16	14.95	0.62^*∗∗*^	0.50	0.74
Poland	22.80^*∗∗*^	19.39	26.81	0.72^*∗∗*^	0.60	0.84
Spain	2.17^*∗∗*^	1.85	2.55	0.34^*∗∗*^	0.20	0.49
Sweden	22.21^*∗∗*^	19.05	25.90	0.56^*∗∗*^	0.44	0.68
Switzerland	9.12^*∗∗*^	7.75	10.73	0.75^*∗∗*^	0.62	0.88
Constant				2.90^*∗∗*^	2.70	3.10

^1^With reference to Netherlands.

^*∗∗*^Significant at 1% level, ^*∗*^significant at 5% level, and ^+^significant at 10% level.

**Table 5:** Per-country analysis for outpatient out-of-pocket payments.

Country	AT	BE	CH	CZ	DE	DK	ES	FR	GR	IT	NL	PL	SE
	OR	OR	OR	OR	OR	OR	OR	OR	OR	OR	OR	OR	OR
*Service use (0 = no; 1 = yes)*													
Current smoker	0.47^*∗∗*^	0.55^*∗∗*^	0.84	0.59^*∗∗*^	0.63^*∗*^	0.65^*∗∗*^	0.87	0.77	0.98	0.74^+^	0.80	0.50^*∗∗*^	0.82
Former smoker	1.23	0.93	1.01	1.12	1.17	1.06	1.31	1.08	1.33^+^	1.15	1.32^*∗*^	0.84	1.30^*∗*^
Light alcohol consumption	1.36	1.20	0.75	0.83	0.61^*∗*^	0.91	0.71^*∗*^	1.24	1.39^*∗∗*^	1.09	1.06	0.68^*∗∗*^	0.90
Heavy alcohol consumption	1.96	0.84	0.50^+^	0.54	0.31^*∗∗*^	0.96	0.84	0.52^*∗*^	0.62^*∗*^	1.04	0.91	0.46^*∗∗*^	1.17
Preobese	0.97	1.06	0.85	1.26	1.01	1.00	0.99	1.83^*∗∗*^	1.00	1.22	0.90	1.47^*∗∗*^	0.85
Obese	0.84	1.04	0.65^+^	1.04	1.07	1.35	0.72	1.59	0.87	1.09	1.02	1.42^*∗*^	0.62^*∗∗*^
*Payments (0 = no; 1 = yes)*													
Current smoker	0.37^*∗*^	1.03	1.31	0.89	1.15	0.78^+^	1.16	1.24	0.98	0.92	0.71^*∗*^	0.58^*∗*^	0.89
Former smoker	0.77	1.24^*∗*^	1.09	1.25	1.15	1.05	1.38	1.05	0.90	1.36^*∗∗*^	0.84	0.65^+^	0.82
Light alcohol consumption	0.81	0.86	2.16^*∗∗*^	1.58^*∗∗*^	1.66^*∗∗*^	1.22	1.00	1.27	0.93	1.27^*∗*^	1.47^*∗*^	1.23	1.28
Heavy alcohol consumption	0.73	0.73^*∗*^	2.62^*∗∗*^	1.48	1.06	1.07	0.93	0.90	0.58^*∗*^	1.17	1.73^*∗*^	1.22	2.16
Preobese	1.01	1.37^*∗∗*^	1.11	0.82	1.06	0.82^*∗*^	1.14	1.02	1.04	0.92	0.89	0.67^+^	1.62^*∗*^
Obese	1.04	1.37^*∗∗*^	0.86	0.66^*∗*^	0.96	0.75^*∗*^	0.61^+^	0.92	0.96	0.77^*∗*^	0.98	0.96	1.13
*High payments (0 = no; 1 = yes)*													
Current smoker	0.87	0.88	0.94	1.93	0.79	1.18	1.26	0.80	1.20	1.06	0.84	1.07	1.22
Former smoker	1.29	0.88	1.09	1.43	1.07	1.53^*∗*^	2.09	1.17	1.11	0.87	1.08	0.90	1.16
Light alcohol consumption	1.33	0.92	0.59^*∗*^	0.56^+^	1.14	0.78	0.98	0.82	0.86	1.08	1.04	—	1.12
Heavy alcohol consumption	0.36	1.18	0.82	3.12	1.09	0.81	2.79	1.12	1.08	0.66	1.49	—	1.11
Preobese	0.92	0.91	0.75^+^	0.56^+^	0.94	0.99	1.33	0.89	1.19	1.00	1.23	2.21	1.11
Obese	0.87	0.93	0.53^*∗*^	0.39^*∗*^	1.27	1.10	1.61	1.85^+^	1.52^*∗*^	1.46^+^	0.55^+^	0.99	0.96

^*∗∗*^Significant at 1% level, ^*∗*^significant at 5% level, and ^+^significant at 10% level.

AT, Austria; BE, Belgium; CH, Switzerland; CZ, Czech Republic; DE, Germany; DK, Denmark; ES, Spain; FR, France; GR, Greece; IT, Italy; NL, Netherlands; PL, Poland; SE, Sweden.

**Table 6:** Per-country analysis for prescribed drug out-of-pocket payments.

	AT	BE	CH	CZ	DE	DK	ES	FR	GR	IT	NL	PL	SE
*Payments (0 = no; 1 = yes)*	OR	OR	OR	OR	OR	OR	OR	OR	OR	OR	OR	OR	OR

Current smoker	0.75	0.91	1.03	0.63^*∗∗*^	0.76^*∗*^	0.92	0.80	0.72^*∗*^	1.06	0.87	0.63^*∗∗*^	0.57^*∗∗*^	0.77^+^
Former smoker	1.18	1.09	1.11	0.87	0.99	1.04	1.21	0.92	1.29^+^	0.85	1.09	0.92	1.04
Light alcohol consumption	1.34	0.89	1.61^*∗∗*^	0.70^*∗∗*^	1.17	0.74	1.00	1.18	1.22^*∗*^	1.08	1.04	0.70^*∗∗*^	0.92
Heavy alcohol consumption	1.93	0.85	1.51	0.56^*∗*^	0.83	0.73	1.14	1.38^+^	0.78	0.93	1.18	0.35^*∗∗*^	1.43
Pre-obese	1.20	1.20	1.12	1.14	1.00	1.15	1.26^+^	0.99	1.05	1.16	0.79^+^	1.51^*∗∗*^	1.01
Obese	1.07	1.54^*∗*^	0.87	1.33^+^	1.33^+^	1.07	0.88	0.87	1.31^*∗*^	0.92	0.78	1.72^*∗∗*^	1.09

*Linear log transformation*	Coef	Coef	Coef	Coef	Coef	Coef	Coef	Coef	Coef	Coef	Coef	Coef	Coef

Current smoker	0.03	0.06	0.05	−0.01	0.16^*∗*^	−0.01	0.12	−0.04	−0.06	0.08	−0.09	−0.09	0.06
Former smoker	0.11	0.13^*∗*^	0.10	−0.05	0.10^+^	0.09	0.34^*∗*^	0.14	0.12^*∗*^	0.06	−0.27	0.07	0.01
Light alcohol consumption	0.00	−0.20^*∗∗*^	−0.22^+^	−0.44^*∗∗*^	−0.11^+^	−0.28^*∗∗*^	−0.13	−0.32^*∗∗*^	−0.07	−0.07	−0.33^+^	−0.33^*∗∗*^	0.02
Heavy alcohol consumption	−0.10	−0.08	−0.39^*∗*^	−0.24^+^	−0.31^*∗*^	−0.49^*∗∗*^	−0.23	−0.40^*∗*^	−0.09	−0.15	−0.55^*∗*^	−0.83^*∗∗*^	0.19
Pre-obese	−0.02	0.04	0.04	0.07	−0.03	0.06	−0.23^+^	0.03	−0.01	−0.06	−0.42	0.14^*∗*^	0.13^*∗∗*^
Obese	0.10	0.00	0.19	0.16^*∗∗*^	0.10	−0.11	−0.03	0.12	0.01	0.04	−0.05	0.28^*∗∗*^	0.20^*∗∗*^

^*∗∗*^Significant at 1% level, ^*∗*^significant at 5% level, and ^+^significant at 10% level.

AT, Austria; BE, Belgium; CH, Switzerland; CZ, Czech Republic; DE, Germany; DK, Denmark; ES, Spain; FR, France; GR, Greece; IT, Italy; NL, Netherlands; PL, Poland; SE, Sweden.
