# Supplementary material for: Patient Payment and Unhealthy Behavior: A Comparison across European Countries
Source: Biomed Res Int. 2017 Feb 5;2017:2615105. doi: 10.1155/2017/2615105 (PMC5316444; doi:10.1155/2017/2615105)
Supplement: Supplementary file 1 — Supplementary Material provides more detailed information about the results. Appendix A1 shows detailed information on the sample characteristics regarding sociodemographic and health status. Appendix A2-4 report the results of uncontrolled models for analysis done in the paper for outpatient and inpatient out-of-pocket payment, as well as for prescirbed drug out-of-pocket payment. [file 2615105.f1.docx]

**Appendix A1: Socio-demographic and health status characteristics**

|  | **Austria** | **Belgium** | **Czech**  **Republic** | **Denmark** | **France** | **Germany** | **Greece** | **Italy** | **Netherland** | **Poland** | **Spain** | **Sweden** | **Switzerland** | **Total** |
| --- | --- | --- | --- | --- | --- | --- | --- | --- | --- | --- | --- | --- | --- | --- |
| Gender |  |  |  |  |  |  |  |  |  |  |  |  |  |  |
| Male (%) | 40.7 | 45.3 | 42.1 | 45.0 | 42.9 | 46.1 | 43.1 | 45.1 | 45.6 | 43.5 | 45.0 | 46.2 | 44.1 | 44.3 |
| Female (%) | 59.3 | 54.7 | 57.9 | 55.1 | 57.1 | 53.9 | 56.9 | 54.9 | 54.5 | 56.5 | 55.0 | 53.8 | 55.9 | 55.7 |
| n | 1341 | 3169 | 2830 | 2616 | 2968 | 2568 | 3243 | 2983 | 2661 | 2467 | 2228 | 2745 | 1462 | 33281 |
| Living with spouse or partner |  |  |  |  |  |  |  |  |  |  |  |  |  |  |
| Yes (%) | 62.9 | 74.4 | 70.6 | 74.5 | 71.3 | 80.8 | 73.1 | 81.7 | 80.3 | 75.8 | 77.7 | 76.8 | 71.1 | 75.2 |
| No (%) | 37.2 | 25.6 | 29.4 | 25.5 | 28.7 | 19.2 | 27.0 | 18.3 | 19.7 | 24.2 | 22.4 | 23.3 | 28.9 | 24.8 |
| n | 1,214 | 3169 | 2830 | 2616 | 2968 | 2566 | 3243 | 2938 | 2661 | 2467 | 2228 | 2744 | 1462 | 33151 |
| Years of education |  |  |  |  |  |  |  |  |  |  |  |  |  |  |
| Mean | 8.7 | 11.7 | 11.7 | 13.1 | 11.2 | 12.5 | 8.5 | 7.9 | 11.1 | 9.2 | 7.3 | 11.2 | 11.4 | 10.5 |
| SD | 4.3 | 3.6 | 3.0 | 3.4 | 4.1 | 3.2 | 4.3 | 4.4 | 3.7 | 3.3 | 5.0 | 3.9 | 4.6 | 4.3 |
| n | 1064 | 3145 | 2817 | 2610 | 2907 | 2555 | 3168 | 2959 | 2633 | 2457 | 2112 | 2714 | 1418 | 32559 |
| Annual household net income(€) |  |  |  |  |  |  |  |  |  |  |  |  |  |  |
| Mean | 26674 | 32165 | 8273 | 42992 | 37633 | 34814 | 18507 | 25545 | 41747 | 6127 | 19980 | 36744 | 56656 | 29005 |
| SD | 19668 | 36945 | 7293 | 29277 | 36925 | 38245 | 19102 | 24286 | 47075 | 6042 | 25806 | 26428 | 51877 | 33320 |
| n | 1341 | 3169 | 2830 | 2616 | 2967 | 2568 | 3243 | 2983 | 2661 | 2467 | 2228 | 2745 | 1462 | 33280 |
| Household real net asset (€) |  |  |  |  |  |  |  |  |  |  |  |  |  |  |
| Mean | 163884 | 253850 | 49158 | 298701 | 354920 | 194137 | 194123 | 277954 | 278524 | 45564 | 275370 | 246304 | 461299 | 233552 |
| SD | 212994 | 306457 | 55593 | 532843 | 746727 | 391723 | 306979 | 378965 | 695141 | 426283 | 633809 | 520058 | 1228892 | 543052 |
| n | 1341 | 3169 | 2830 | 2616 | 2967 | 2568 | 3243 | 2983 | 2661 | 2467 | 2228 | 2745 | 1462 | 33280 |
| Household size |  |  |  |  |  |  |  |  |  |  |  |  |  |  |
| Mean | 1.9 | 2.1 | 2.1 | 1.9 | 2.1 | 2.1 | 2.4 | 2.6 | 2.1 | 3.0 | 2.6 | 1.9 | 2.1 | 2.2 |
| SD | 0.9 | 0.9 | 1.0 | 0.7 | 1.1 | 0.8 | 1.1 | 1.1 | 0.8 | 1.7 | 1.1 | 0.7 | 0.9 | 1.1 |
| n | 1192 | 3169 | 2830 | 2616 | 2968 | 2568 | 3243 | 2983 | 2661 | 2467 | 2228 | 2745 | 1462 | 33132 |
| Self-perceived health |  |  |  |  |  |  |  |  |  |  |  |  |  |  |
| Very good (%) | 29.0 | 28.4 | 19.1 | 51.8 | 20.7 | 21.8 | 36.4 | 20.2 | 27.6 | 7.7 | 13.9 | 40.3 | 45.5 | 27.5 |
| Less than very good (%) | 71.0 | 71.6 | 80.9 | 48.2 | 79.3 | 78.2 | 63.6 | 79.9 | 72.4 | 92.4 | 86.1 | 59.7 | 54.5 | 72.5 |
| n | 1340 | 3168 | 2823 | 2610 | 2923 | 2562 | 3230 | 2977 | 2646 | 2457 | 2225 | 2741 | 1455 | 33157 |
| Number of chronic disease |  |  |  |  |  |  |  |  |  |  |  |  |  |  |
| Mean | 1.2 | 1.3 | 1.3 | 1.3 | 1.2 | 1.1 | 1.2 | 1.5 | 0.9 | 1.6 | 1.3 | 1.2 | 0.8 | 1.2 |
| SD | 1.2 | 1.3 | 1.3 | 1.3 | 1.2 | 1.2 | 1.3 | 1.4 | 1.1 | 1.5 | 1.3 | 1.3 | 1.0 | 1.3 |
| n | 1191 | 3167 | 2822 | 2611 | 2920 | 2561 | 3214 | 2977 | 2644 | 2457 | 2225 | 2741 | 1455 | 32985 |
| Number of symptoms |  |  |  |  |  |  |  |  |  |  |  |  |  |  |
| Mean | 1.8 | 1.9 | 2.0 | 1.6 | 1.9 | 1.6 | 1.5 | 1.9 | 1.3 | 2.7 | 1.8 | 1.7 | 1.3 | 1.8 |
| SD | 1.8 | 1.9 | 2.0 | 1.8 | 1.9 | 1.7 | 1.8 | 2.0 | 1.6 | 2.3 | 2.0 | 1.7 | 1.5 | 1.9 |
| n | 1340 | 3168 | 2823 | 2609 | 2920 | 2562 | 3237 | 2976 | 2646 | 2456 | 2225 | 2739 | 1455 | 33156 |

**Table A2: Sequential logit for outpatient out-of-pocket payment (uncontrolled model)**

|  | **Either used or not** | | | **Either paid or not** | | | **Either paid high or low amount** | | |
| --- | --- | --- | --- | --- | --- | --- | --- | --- | --- |
|  | OR | 95%CI | 95%CI | OR | 95%CI | 95%CI | OR | 95%CI | 95%CI |
| Daily smoking |  |  |  |  |  |  |  |  |  |
| Current smoker | 0.57*** | 0.53 | 0.62 | 0.90*** | 0.83 | 0.96 | 0.99 | 0.89 | 1.10 |
| Former smoker | 1.08* | .99 | 1.17 | 1.11*** | 1.05 | 1.18 | 1.09* | 0.99 | 1.19 |
| Alcohol consumption |  |  |  |  |  |  |  |  |  |
| Not excessive alcohol use | 0.75*** | 0.69 | 0.81 | 1.72*** | 1.63 | 1.82 | 0.93 | 0.89 | 1.10 |
| Excessive alcohol use | 0.63*** | 0.55 | 0.72 | 1.24*** | 1.11 | 1.39 | 0.99 | 0.82 | 1.18 |
| Body Mass Index |  |  |  |  |  |  |  |  |  |
| Overweight | 1.17*** | 1.09 | 1.26 | .90*** | 0.85 | 0.95 | 1.01 | 0.93 | 1.10 |
| Obese | 1.46*** | 1.32 | 1.61 | 0.77*** | 0.71 | 0.82 | 1.15 | 1.02 | 1.28 |

*** Significant at 1% level, ** significant at 5% level, * significant at 10% level

**Table A3: Sequential logit for inpatient out-of-pocket payment (uncontrolled model)**

|  | **Either used or not** | | | **Either paid or not** | | | **Either paid high or low amount** | | |
| --- | --- | --- | --- | --- | --- | --- | --- | --- | --- |
|  | OR | 95% CI | 95% CI | OR | 95% CI | 95% CI | OR | 95% CI | 95% CI |
| Daily smoking |  |  |  |  |  |  |  |  |  |
| Current smoker | 0.92* | 0.84 | 1.01 | 0.68*** | 0.57 | 0.81 | 1.01 | 0.76 | 1.34 |
| Former smoker | 1.47*** | 1.37 | 1.59 | 0.86** | 0.75 | 0.99 | 1.12 | 0.91 | 1.38 |
| Alcohol consumption |  |  |  |  |  |  |  |  |  |
| Not excessive alcohol use | 0.60*** | 0.56 | 0.64 | 1.74*** | 0.1.53 | 1.98 | 0.88 | 0.72 | 1.08 |
| Excessive alcohol use | 0.48*** | 0.42 | 0.56 | 1.34** | 1.004 | 1.81 | 1.05 | 0.65 | 1.69 |
| Body Mass Index |  |  |  |  |  |  |  |  |  |
| Overweight | 0.97 | 0.90 | 1.04 | 0.91 | 0.80 | 1.05 | 0.96 | 0.78 | 1.18 |
| Obese | 1.18*** | 1.08 | 1.28 | 0.75*** | 0.63 | 0.88 | 0.88 | 0.68 | 1.15 |

*** Significant at 1% level, ** significant at 5% level, * significant at 10% level

**Table A4: Two part model for prescribed drug out-of-pocket payments (uncontrolled model)**

|  | *First part: logit*  **Either paid or not** | | | *Second part: log transformation*  **The amount of OOP(log)** | | |
| --- | --- | --- | --- | --- | --- | --- |
|  | OR | 95%CI | 95%CI | coefficient | 95%CI | 95%CI |
| Daily smoking |  |  |  |  |  |  |
| Current smoker | 0.83*** | 0.78 | 0.88 | -0.01 | -0.06 | 0.03 |
| Former smoker | 0.97 | 0.92 | 1.02 | 0.16*** | 0.12 | 0.20 |
| Alcohol consumption |  |  |  |  |  |  |
| Not excessive alcohol use | 0.92*** | 0.87 | 0.96 | -0.31*** | -0.35 | -0.27 |
| Excessive alcohol use | 0.64*** | 0.54 | 0.70 | -0.31*** | -0.39 | -0.23 |
| Body Mass Index |  |  |  |  |  |  |
| Overweight | 1.20*** | 1.14 | 1.26 | 0.02 | -0.015 | 0.062 |
| Obese | 1.48*** | 1.39 | 1.58 | 0.17*** | 0.12 | 0.22 |

*** Significant at 1% level, ** significant at 5% level, * significant at 10% level
